# Supplementary material for: Characterization of Molecular Chaperone GroEL as a Potential Virulence Factor in Cronobacter sakazakii
Source: Foods. 2023 Sep 12;12(18):3404. doi: 10.3390/foods12183404 (PMC10528849; doi:10.3390/foods12183404)
Supplement: Supplementary file 1 [file foods-12-03404-s001.zip › foods-2586677-supplementary.pdf]

Supplementary Data

Characterization of molecular chaperone GroEL as a potential virulence factor in *Cronobacter sakazakii*

Dong-dong Zhu[a], Yu-fei Fan[a], Xiao-yi Wang[a], Ping Li[a], Ya-ping Huang[a], Jing-bo Jiao[a], Chu-min Zhao[a], Yue Li[a], Shuo Wang[a,b,], Xin-jun Du[a,*]

[a] *State Key Laboratory of Food Nutrition and Safety, College of Food Science and Engineering, Tianjin University of Science and Technology, Tianjin 300457, China*

[b] *Tianjin Key Laboratory of Food Science and Health, School of Medicine, Nankai University, Tianjin 300071, China*

* Corresponding author: State Key Laboratory of Food Nutrition and Safety, College of Food Science and Engineering, Tianjin University of Science and Technology, Tianjin 300457, China

Tel.: +86 22 60912484; Fax: +86 22 60912484

E-mail address: xjdu@tust.edu.cn (XD)

Corresponding author: State Key Laboratory of Food Nutrition and Safety, College of Food Science and Engineering, Tianjin University of Science and Technology, Tianjin 300457, China

Tel.: +86 22 60912484; Fax: +86 22 60912484

E-mail address: s.wang@tust.edu.cn; wangshuo@nankai.edu.cn (SW)

Figure S1. The quaternary structure of GroEL in various fractions. Based on the content of GroEL in the cytoplasm, the proportion of GroEL in other fractions is calculated through gray-scale scanning, so as to calculate the loading quantity of other fractions of protein samples. Lane 1, cytoplasm (10 μg); Lne 2, cytoplasmic membrane (31 μg); Lane 3, outer membrane (125 μg); Lane 4, periplasm (19 μg);Lane 5, ultracentrifuged supernatant (64 μg); Lane6, OMV (13 μg).

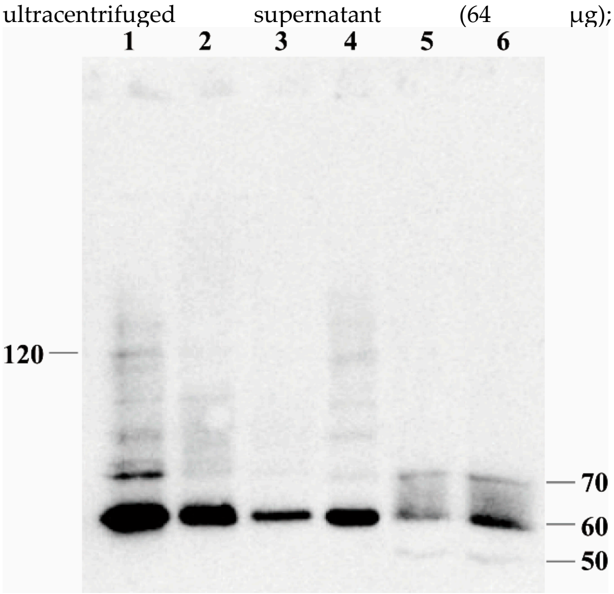

Figure S2. (A) Bacterial growth curve (37 °C); (B) MTT experiment assays of the viability vitality of logarithmic-phase bacteria which is in logarithmic phase. ns: no significant difference.

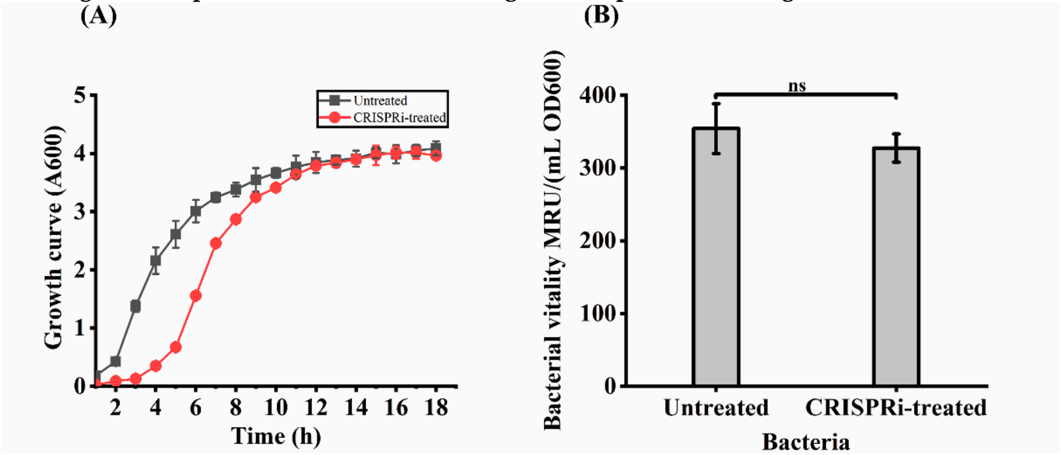

Figure S3. Determination of the production of LPS, and two groups of LPS samples with the equal amounts were loaded. (A) Quantification of LPS production; (B) Silver staining assays of LPS components. Lane1, Untreated group; Lane 2, CRISPRI-treated group. ns: no significant difference.

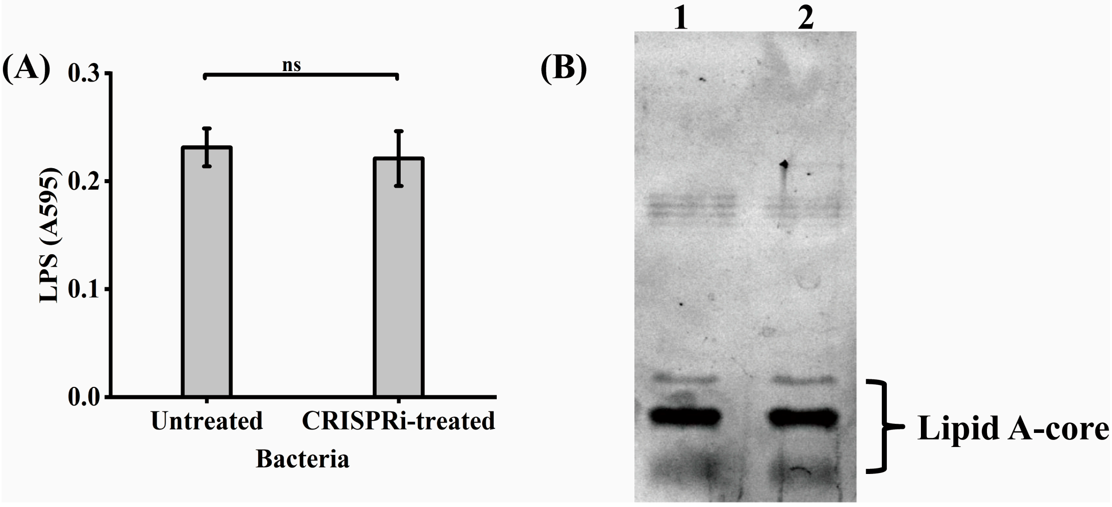

*

Extraction and quantification of bacterial LPS were performed as Kim's methods [14]. Briefly, all bacterial strains were cultured to OD600 of 1.0, followed by extracting an equivalent volume of LPS from the bacterial suspension of each group with phenol solution (68 °C). The crude LPS were digested with DNAase and RNAase to acquire purified LPS. NaIO4 and purpald reagent were used to analyze the LPS of different groups. The absorbance was measured for each group at OD595.

Table S1. Primers used in this study

Primers	
F1	AGTCAGGCACCGTGTATGGCAGCTAAAGACGTAAAATTCGG
R1	GCCGCCGGCTTCCATTCAGTGGTGGTGGTGGTGG
F2	ATGGAAGCCGGCGGCACC
R2	ACACGGTGCCTGACTGCGTTAGC
F3	GGACACGCCGTCTTTGGTGAGTTTTAGAGCTAGAAATAGCAAGTTAAAATAAGGCTAG TCC
R3	ACTAGTATTATACCTAGGACTGAGCTAGCTGTCAAG
F(GAPDH)	AGGTCGGAGTCAACGGATTT
R(GAPDH)	TGGAAGATGGTGATGGGATTT
F(CLDN1)	GGATTTACTCCTATGCCGGCGACAACA
R(CLDN1)	CTCTGCGACACGCAGGACATCCA
F(OCLN)	ATGAGACAGACTACACAACTGG
R(OCLN)	TTGTATTCATCAGCAGCAGC
F(ZO-1)	AAGTCACACTGGTGAAATCC
R(ZO-1)	CTCTTGCTGCCAAACTATCT
F(ZO-2)	GCCGCTAAGAGCACAGCAA
R(ZO-2)	TCCCCACTCTGAAAATGAGGA
